# The Benefit of Gratitude: Trait Gratitude Is Associated With Effective Economic Decision-Making in the Ultimatum Game

**DOI:** 10.3389/fpsyg.2021.590132

**Published:** 2021-04-20

**Authors:** Gewnhi Park, Charlotte vanOyen-Witvliet, Jorge A. Barraza, Benjamin U. Marsh

**Affiliations:** ^1^Psychology Department, Hope College, Holland, MI, United States; ^2^Department of Psychology, University of Southern California, Los Angeles, CA, United States; ^3^Department of Psychology, University of Tampa, Tampa, FL, United States

**Keywords:** emotion, decision-making, the ultimatum game, trait gratitude, state gratitude

## Abstract

The current research investigated the role of gratitude in economic decisions about offers that vary in fairness yet benefit both parties if accepted. Participants completed a trait/dispositional gratitude measure and then were randomly assigned to recall either an event that made them feel grateful (i.e., induced gratitude condition) or the events of a typical day (i.e., neutral condition). After the gratitude induction task, participants played the ultimatum game (UG), deciding whether to accept or reject fair offers (i.e., proposer: responder ratio $5:5) and unfair offers (i.e., proposer: responder ratios of $9:1, $8:2, or $7:3) from different proposers. Results showed that trait gratitude was positively correlated with respondents’ acceptance of unfair offers. However, experimentally induced momentary gratitude did not influence acceptance of unfair offers. The trait or disposition to be grateful involves the enduring capacity across different types of situations and benefactors to see the good that is present, even when that benefit is small. Accordingly, dispositional gratitude – but not momentarily induced gratitude – was associated with a greater propensity to accept even the small benefits within unfair offers which otherwise pose barriers to making the effective economic decision of accepting offers regardless of their relative size.

## Introduction

Gratitude involves seeing and appreciating benefits given by others to oneself and has been linked to generosity (McCullough et al., 2002). The disposition to be grateful plays an important role in developing and maintaining social engagement by motivating people to behave in ways that also benefit others (McCullough et al., 2008). Further, gratitude is associated with other positive traits and dispositions, such as agreeableness (McCullough et al., 2002), forgivingness, patience, happiness, and hope (Witvliet et al., 2018a). Like other positive emotions, gratitude broadens the scope of one’s cognition and behaviors, which allows for developing psychological and social resources (Fredrickson, 2013).

Converging evidence suggests that gratitude may play an important role in decision-making. Some researchers have shown that gratitude facilitates more rational economic decision-making (DeSteno et al., 2010, 2014; Dickens and DeSteno, 2016). Specifically, participants who experienced an induced gratitude state demonstrated increased patience, which in turn allowed them to choose larger long-term benefits over small immediate benefits in the delay discounting task (DeSteno et al., 2010; Dickens and DeSteno, 2016). Moreover, people with higher levels of state gratitude were more likely to help those who requested assistance even if it was costly for a short period (Bartlett and DeSteno, 2006). More recently, it was found that individuals with higher (vs. lower) dispositional gratitude were more likely to make risk aversion decisions – choosing to accept an assured present good rather than risk losing it for a potentially better outcome later (Zhang et al., 2020). Not only that but also experimentally induced gratitude led to more risk aversion decision-making (Zhang et al., 2020). These findings suggest that gratitude may shape decision-making processes. In the current research, we assessed whether gratitude as a dispositional trait or temporarily induced state would facilitate optimal ultimatum game (UG) economic decisions – defined as accepting unfair offers of a small amount of money knowing that the other person will benefit even more.

### The Ultimatum Game

The UG task offers a laboratory model of economic decision-making, which has been widely used to study how people respond to violations of the fairness norm (van’t Wout et al., 2010). In the UG task, the proposer is given a sum of money and makes an offer to the responder as to how to split the money between the two of them (Sanfey et al., 2006; Scheres and Sanfey, 2006). Then, the responder makes a decision about whether to accept or reject the offer. When the responder accepts the offer, the money will be split between the two players according to the proposer’s offer. When the responder rejects the offer, neither the proposer nor the responder receives anything. Therefore, the rational response for the responder is to accept any offer because any monetary reward is preferable to none.

Some offers are considered fair in that the money is evenly split between the proposer and the responder. However, other offers are not equitable. In these unfair offers, the proposer receives substantially more money than the responder. Responders frequently reject unfair offers, even if it means they will not receive any money (Pillutla and Murnighan, 1996; Sanfey et al., 2003; Tabibnia et al., 2008). Some have construed the rejection of unfair offers as “altruistic punishment” for norm violation (Fehr and Rockenbach, 2004; Frith and Singer, 2008; Phelps, 2009). According to the norm compliance framework, fair sharing is considered to be a social norm that people inherently prefer, even at the expense of their own monetary sacrifice (Gächter et al., 2017). Thus, one possible explanation for the rejection of unfair offers is that the responders experience an aversive emotion, such as anger, in response to unfair offers (Pillutla and Murnighan, 1996; Fehr and Rockenbach, 2004; Frith and Singer, 2008; Robson et al., 2020). As a result, the responders are willing to punish the proposers who made unfair offers by depriving them from getting a greater share of the money, even at the cost of forfeiting small monetary gain for themselves.

Extensive behavioral and physiological evidence aligns with the interpretation that negative emotions, such as anger, evoked by fairness norm violations are associated with the rejection of unfair offers (Pillutla and Murnighan, 1996; van’t Wout et al., 2006, 2010; Robson et al., 2020). For example, one influential neuroimaging study (Sanfey et al., 2003) has shown that unfair offers elicited the activation of the bilateral insula implicated in negative emotions such as anger (Damasio et al., 2000) and disgust (Phillips et al., 1997), as well as motivational states such as pain (Derbyshire et al., 1997), hunger (Denton et al., 1999), and thirst (Tataranni et al., 1999). Furthermore, people with stronger insula activation were more likely to reject unfair offers (Sanfey et al., 2003). Unfair offers also elicited greater sympathetic nervous system activation, indicated by higher skin conductance (sweat) activity (van’t Wout et al., 2006). This sweat response pattern was apparent only when human counterparts, not computer counterparts, proposed offers, indicating the response is to the action of a person rather than the receipt of a lower amount. Also, an event-related potential (ERP) study has revealed that unfair offers elicited greater feedback negativity (FN), an ERP component evoked around 300–500 ms (Osinsky et al., 2014). Previous research has shown that FN reflects “good vs. bad evaluation” that resulted from dopagmergic signaling in the medial frontal cortex (Gehring, 2002; Hajcak et al., 2006; Kaltwasser et al., 2016). As such, the stress responses evoked by perceiving another person’s violation of the fairness norm may account for why respondents make the irrational economic decision of rejecting unfair offers, as is frequently observed in the UG.

Alternatively, another line of research has suggested that the rejection of unfair offers is also predicted by assertiveness (Yamagishi et al., 2012; Kaltwasser et al., 2016). According to the status defense model (Yamagishi et al., 2012; Kaltwasser et al., 2016), more assertive people avoid compliance when treated unfairly and instead signal their control by rejecting unfair offers at the expense of their own monetary gains. In other words, assertive people reject unfair offers because they are unwilling to be viewed as weak, inferior, or undeserving of fair treatment (Kaltwasser et al., 2016). This research suggests the possibility that individual differences in personality traits play an important role in determining the rejection of unfair offers.

A further line of research points to the complexity of emotion, sociality, and cognition by examining psychiatric conditions in relation to rejecting or accepting unfair offers (Robson et al., 2020). The typical pattern of rejecting unfair offers has been found in people with unipolar depression and bipolar mood disorders, which aligns with having negative responses to difficult social interactions. By contrast, people with anxiety disorders, autism spectrum disorder, and schizophrenia have been found to accept more unfair offers, perhaps because anxiety disorders can involve avoidance of negative situations including social confrontation, autism spectrum disorder involves challenges with theory of mind, and schizophrenia involves dysregulation among cognitive, affective, and behavioral responses (Robson et al., 2020). People who score higher on psychopathy have also been found to accept more unfair offers (Osumi and Ohira, 2010). This may be due to a variety of reasons, including immediate self-interest for reward, amygdala differences that impact learning in relation to negative or positive stimuli, connections to the caudate and orbitofrontal cortex which are involved in predicting outcomes, and other executive functions that may be impacted by abnormalities of the prefrontal cortex (see Nickerson, 2014 meta-analysis).

Finally, we examined a line of research in which emotion regulation increases acceptance of offers that are unfair – those that disproportionately benefit the proposer yet nevertheless also benefit the responder. This literature is important for the current study, which assessed whether individual differences in trait gratitude or a temporarily induced state of gratitude would influence the rejection of unfair offers in the UG. According to the norm compliance model, responders are willing to forego the benefit of an immediate reward in order to punish proposers who make unfair offers by rejecting their offers. This deprives the unfair proposers from getting a greater share of the offer, but also forfeits responders’ own monetary gain due to their own anger or frustration driven response (Sanfey et al., 2006). Down-regulating negative emotions may be critical in facilitating the acceptance of unfair offers. Greater acceptance of unfair offers has been associated with neuroimaging evidence of increased activity in the right ventrolateral prefrontal cortex, implicated in emotion regulation (Tabibnia et al., 2008). Previous research also showed that implementing an emotion regulation strategy significantly increased the acceptance rate of unfair offers (van’t Wout et al., 2010). When participants were instructed to reappraise their emotions while receiving unfair offers, they were more likely to accept these unfair offers – a strategy that yielded more economic gain for the responders – compared to when they did not engage in emotion regulation strategies. Thus, emotion regulation strategies allowed people to down-regulate or override negative emotions associated with violations of the fairness norm, thereby lessening the motivation to reject unfair offers. Furthermore, emotion regulation may allow people to see the positive aspect of accepting offers (e.g., receiving some money, generously accommodating a greater payout to someone who may be in need of money). Thus, regulating negative emotions associated with fairness norm violation and recognizing the positive effects of accepting offers may play an important role in facilitating more effective economic decisions in the UG task. With respect to the current study, gratitude may serve to down-regulate negative emotions evoked by violation of the fairness norm, in part by prompting positive appraisals and behaviors that allow all parties to benefit (Dickens and DeSteno, 2016).

The status defense model has been advanced to account for the finding that more assertive people have higher rates of rejecting unfair offers (Yamagishi et al., 2012; Kaltwasser et al., 2016). Defending or asserting one’s status may be especially important when one feels threatened by unfair treatment or when one lacks confidence in one’s self-identity. People high in gratitude likely are less concerned with defending their status. Research has shown trait gratitude to be associated with self-esteem (Bartlett et al., 2020), a secure sense of self-identity, being less influenced by external factors, and the capacity to behave in ways that are consistent with personal beliefs and values (Wood et al., 2008, 2010). Dispositional gratitude is also associated with agreeableness, empathy, forgiveness, and generosity, as well as inversely associated with envy and anger (see McCullough et al., 2002). In terms of the current study, individuals with high trait gratitude may be less likely to engage in defense of their status or attempts to promote self-assertiveness by rejecting unfair offers and more likely to see the benefit of accepting unfair offers for both parties involved. To the extent that induced episodes of gratitude also activate effective emotion-regulation and reduce defensiveness, state gratitude may also increase acceptance of unfair offers, which – although inequitable – allow both the proposer and responder to benefit.

### Overview of Current Gratitude Study

In the current research, we examined whether gratitude would be associated with greater acceptance of unfair offers in the UG. When responders accept offers, a monetary benefit goes to both parties – regardless of whether the money is fairly divided or whether the proposer unfairly receives a disproportionately greater amount of the money compared to the responder. Thus, accepting (vs. rejecting) unfair offers represents more optimal economic decision-making because receiving something is greater than receiving nothing (e.g., Robson et al., 2020).

Gratitude, when expressed as a disposition or characteristic trait, involves a pattern of perceiving and valuing the good that is present, including small benefits and even in difficult situations. This may contribute to the direct correlation found between the social virtues of gratitude and forgiveness (McCullough et al., 2002; Witvliet et al., 2018a). Further work highlights that in the context of an interpersonal offense, a gratitude-rooted benefit-focused reappraisal induction increased forgiveness, calmed and elevated positive emotion, and increased heart rate variability suggesting parasympathetic nervous system activation (Witvliet et al., 2010). Accordingly, gratitude may bolster the capacity to overcome perceptions of injustice when unequal offers are received. A distinct line of research links gratitude to greater self-esteem (Bartlett et al., 2020) and a more secure self-identity that can withstand external forces in order to behave in line with personal values (Wood et al., 2008, 2010). Accordingly, these findings suggest that gratitude may also diminish the tendency to defend one’s status in the face of unfair offers.

Thus, we hypothesized that when receiving unfair offers in the UG task, people with greater trait gratitude would be more likely to accept the benefit of economic gain even though it is proportionally smaller than what the proposer stands to gain. Additionally, we tested the hypothesis that induced state episodes of gratitude would also be associated with higher rates of accepting unfair offers.

## Materials and Methods

### Ethics

Written informed consent was obtained from the individual participants for the publication of any potentially identifiable images or data included in this article.

### Participants

Seventy undergraduate students successfully completed the study for partial course credit.^1^ The questionnaire data from one participant was lost due to a computer error, resulting in 69 participants (44 women; mean age = 19 years). The local ethics committee approved the study and all participants provided written informed consent after the procedures had been fully explained, in accordance with the Declaration of Helsinki.

### Procedure

Sixty-nine participants were randomly assigned to one of the two conditions: grateful or neutral. Participants were asked to recall an event that made them feel grateful (induced gratitude condition), or the events of a typical day (neutral condition). Participants were then instructed to spend 5-min writing about the assigned topic in detail. Before and after the mood induction condition, participants completed a measure of state gratitude created by Tsang (2007), which required them to indicate, on a seven-point scale the extent to which they felt pleased, grateful, indebted, happy, resentful, thankful, annoyed, appreciative, obligated, upset, sympathetic, and angry. The seven-point scale ranged from 1 to 7 (1 = feeling very little of this emotion, 7 = feeling a lot of this emotion). To create a composite measure of self-reported grateful emotion we combined the rates of adjectives *grateful*, *appreciative*, and *thankful* reported (Cronbach’s *α* = 0.85 before the induction; Cronbach’s *α* = 0.87 after the induction).

#### The Ultimatum Game

**Figure 1:**
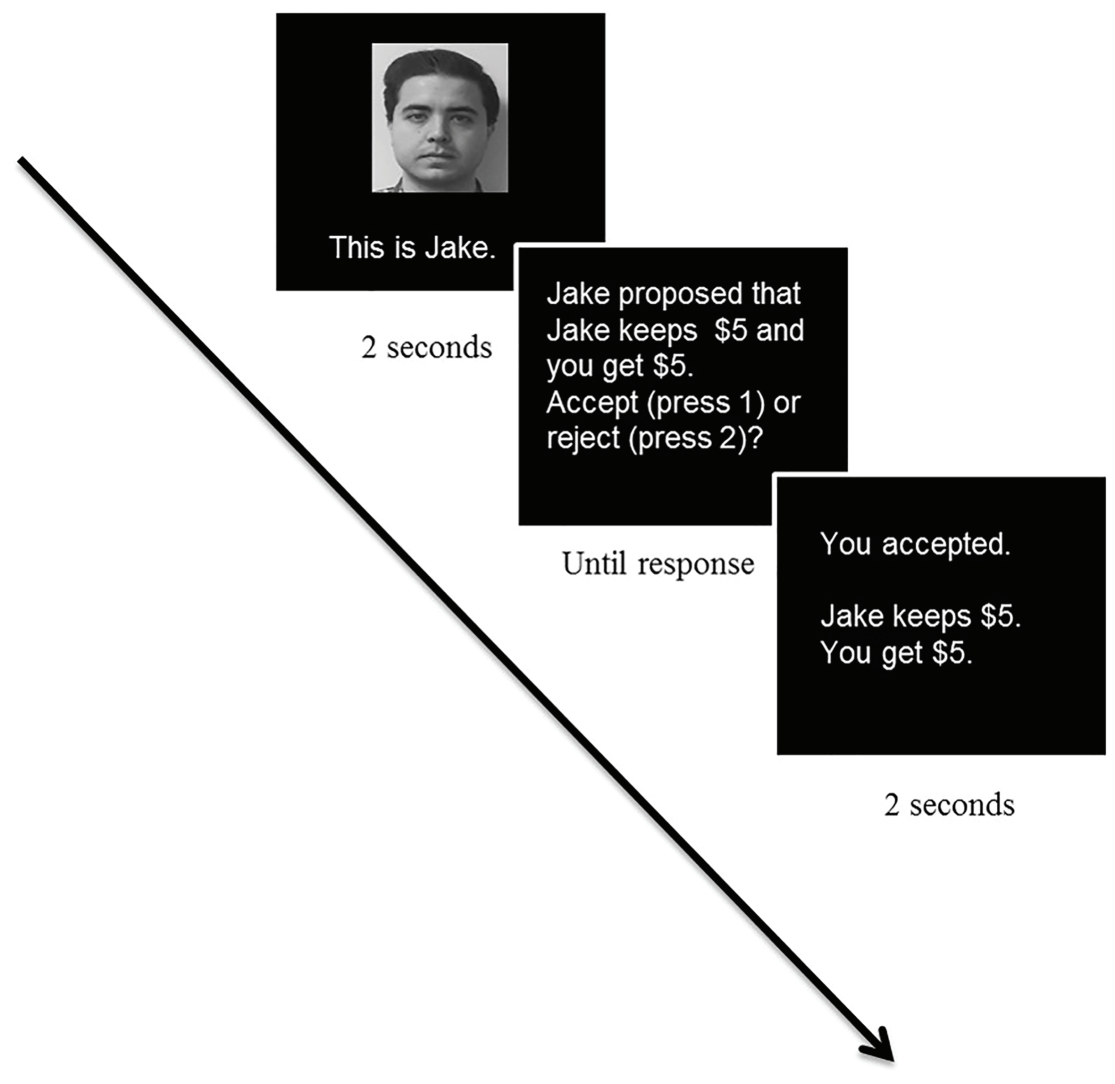
Sample trial in the Ultimatum Game Task. Adapted from Park et al. (2019).

After the gratitude induction task, participants played a modified version of the UG that was adapted from van’t Wout et al. (2010). The UG was programmed in E-prime software (Psychology Software Tools, Pittsburgh). Before starting the experiment, participants first read the instructions and completed three practice trials to ensure that participants fully understood the game. On each round, participants were first presented with a picture of their human opponent, after which the proposal was presented, and participants could respond by pressing a button to accept or reject the offer. There were a total of 24 rounds in which each of the participants played a role as a responder (see Figure 1 for an example of a full trial).

Twenty-four rounds consisted of six fair offers ($5 to each player) and 18 unfair offers defined as offering the participant less than half of the money. The unfair set consisted of six offers of $3, six offers of $2, and six offers of $1. We did not include $4 offers because $4 offers are so close to a fair offer that they are generally perceived as fair and thus frequently accepted. The offers were made by male partners, and the order of partners and the pictures associated with each offer was randomized. Participants were not informed of the total number of rounds in advance. The instructions emphasized that the different partners in the game would play the game independently of each other, and participants were told the games would be played with the set of partners they saw. To encourage participants to make decisions seriously, participants were told they would be paid 5% of the total amount of money earned in the game in addition to course credit. Upon the completion of the UG task, participants were instructed to complete the Gratitude Questionnaire-6 (GQ-6; McCullough et al., 2002). This six-item scale is widely used to assess gratitude as a unidimensional trait (McCullough et al., 2002). These items are rated on a seven-point scale ranging from 1 (*strongly disagree*) to 7 (*strongly agree*). The Cronbach’s *α* coefficient was 0.87.

## Results

### Emotion-Manipulation Check

Measures of state gratitude pre and post gratitude induction task were compared to check the induction manipulation. In order to confirm the success of the manipulation, we conducted a 2 (Induction Condition: neutral, grateful) × 2 (Measured Time: before the induction, after the induction) mixed analysis of variance, with the second factor being repeated. As expected, there was a significant interaction between induction condition and measured time, *F*(1, 67) = 18.62, *p* < 0.001, $\eta_{p}^{2}$ = 0.22. Planned comparisons revealed that people who were induced to feel gratitude reported significantly more elevated feelings of gratefulness after the induction (*M* = 17.6, *SD* = 3.2) compared to before the induction in the gratitude condition (*M* = 15.8, *SD* = 4.1), *t*(34) = −5.11, *p* < 0.01, *d* = 0.49. However, there was no difference in state gratitude for people in the neutral induction condition (*p* = 0.89).^2^

### The Ultimatum Game

**Figure 2:**
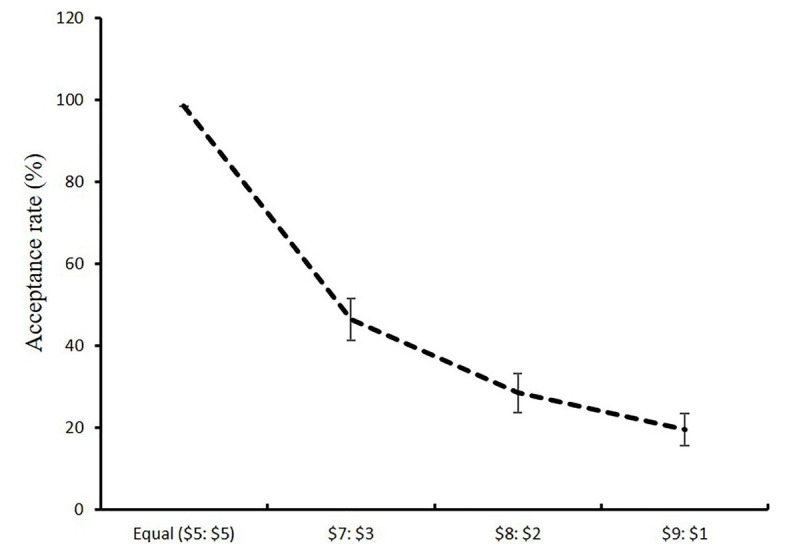
Percentage of acceptance of the different offers.

Across all conditions fair offers ($5) were always accepted and, as is generally seen in the UG, acceptance rates decreased as the offers became progressively more unfair (van’t Wout et al., 2010; see Figure 2): $5-$5: *M* = 98.6% (*SD* = 6.8); $7-$3: *M* = 46.4% (*SD* = 43.0); $8-$2: *M* = 28.6% (*SD* = 39.6); $9-$1: *M* = 19.5% (*SD* = 32.8; See Table 1). We hypothesized that: (a) high trait gratitude would be associated with more frequent acceptance of unfair offers, and (b) participants who experienced higher levels of state gratitude would more frequently accept unfair offers.

**Table 1:** Acceptance rates of four offers as a function of the induction conditions.

	Grateful condition	Neutral condition
*N*	35	35
$5-$5	100 (0)	97.14 (9.47)
$7-$3	43.33 (45.05)	49.52 (41.32)
$8-$2	26.19 (40.48)	30.95 (39.63)
$9-$1	17.62 (35.69)	21.43 (30.13)

Standard deviations in parentheses.

**Figure 3:**
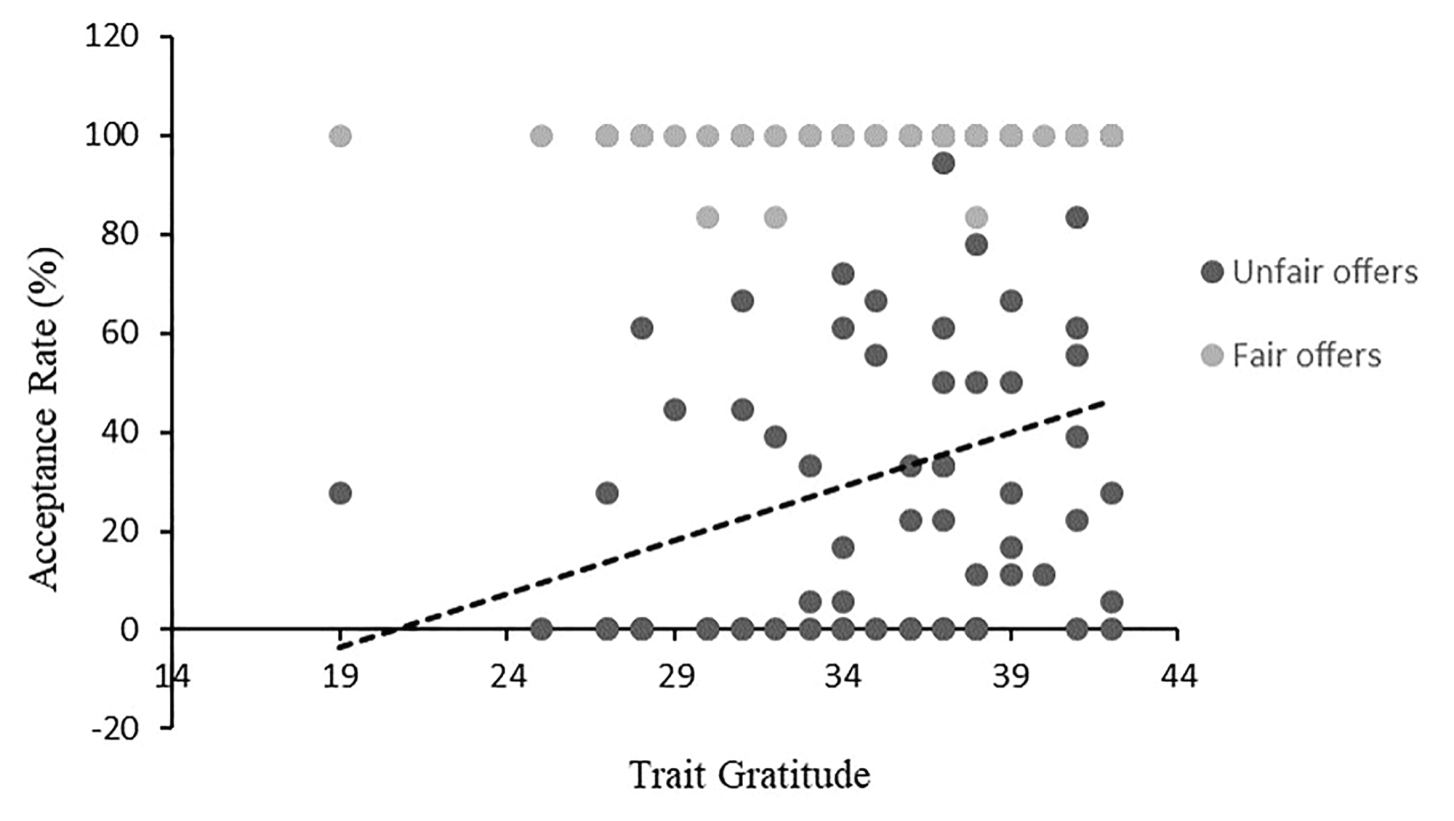
Percentage of acceptance of fair (*r* = 0.09, *p* = 0.49 for $5-$5) and unfair (*r* = 0.32, *p* < 0.01 for unfair offers averaging $7-$3, $8-$2, $9-$1) offers as a function of trait gratitude.

To examine the effect of trait gratitude on acceptance rates of unfair offers, we conducted the Quade test, one of the most frequently cited nonparametric alternatives to repeated measures analysis of covariance (Quade, 1967; Rheinheimer and Penfield, 2001) on acceptance rates of four conditions (Proposer: Respondent Ratios of $5:$5, $7:$3, $8:$2, $9:$1) with trait gratitude (GQ-6) scores as a covariate. As expected, a significant two-way interaction between different offers and trait gratitude was observed, *F*(3, 272) = 82.5, *p* < 0.001. There were significantly positive relationships between trait gratitude and the acceptance rates of unfair offers (*r* = 0.27, *p* < 0.03 for $9:$1; *r* = 0.28, *p* < 0.03 for $8:$2; *r* = 0.28, *p* = 0.02 for $7:$3; see Figure 3 for unfair offers averaged across $9:$1, $8:$2, $7:$3).^3^ Trait gratitude was unrelated to acceptance rates for the fair offer condition ($5:$5, *r* = 0.09, *p* = 0.49). Therefore, consistent with our predictions, participants with high trait gratitude were more likely to accept the unfair offers, regardless of how unfair they were.

To examine the effect of induced gratitude on acceptance rates of unfair offers, we used the Mann-Whitney U test, which is commonly used to analyze the results for UGs because the data often violate the normality assumption. There was no statistical difference between two groups in acceptance rates for unfair offers (*U* = 454, *N*_1_ = 31, *N*_2_ = 33, *p* = 0.43, two-tailed). Thus, contrary to the hypothesis, higher state gratitude did not produce more acceptance of unfair offers.

## Discussion

In the current research, we examined whether trait and state gratitude were each associated with more effective economic decision-making in the UG by accepting unfair offers that nevertheless benefit oneself. As predicted, high trait gratitude was associated with more acceptance of unfair offers in which the proposer kept more money than would be given to the responder (i.e., $9:1, $8:2, or $7:3). Further, people with high gratitude dispositions accepted a greater proportion of unfair offers regardless of how unfair they were. That is, highly grateful people accepted even the smallest benefit proposed. This fits with the inverse relationship gratitude has with envy and materialism, as well as the direct relationship of gratitude with generosity (McCullough et al., 2002). However, although the manipulation to induce elevated state gratitude was successful, higher state gratitude after the mood induction did not elevate acceptances of unfair offers. Thus, the current study provided evidence that trait, but not state, gratitude was associated with more optimal economic decision-making that yielded greater monetary profits.

Gratitude involves the capacity to recognize the good that is present – even when it is small, and even when it occurs in otherwise difficult or unfair situations. Particularly in unfair interactions, gratitude may afford the ability to recognize both the problem at hand as well as the presence of benefits or possibilities. Accordingly, gratitude would facilitate wise decision-making rather than reactive responses. Gratitude is associated with emotion-regulation, likely down-regulating stress reactivity that occurs when the fairness norm is violated (e.g., van’t Wout et al., 2006) and when remembering a prior interpersonal injustice (Witvliet et al., 2010). Furthermore, gratitude involves positive appraisals of even very small benefits that are present, thereby allowing people to engage in better decision-making with more beneficial outcomes for all parties involved (Dickens and DeSteno, 2016).

People who exhibit higher trait gratitude have been found to show several features that may catalyze greater acceptance of unfair offers. One line of research has linked gratitude to greater self-esteem (Bartlett et al., 2020), secure self-identity, and authenticity that is less influenced by external influences such as others’ views or reputations (see Wood et al., 2008, 2010). Thus, more grateful people may not behave in line with the status defense model in which people with more assertive personalities have been found to reject unfair offers in part because they have an aversion to being viewed as inferior (Yamagishi et al., 2012; Kaltwasser et al., 2016). Moreover, previous research has shown that people with high trait gratitude tend to have positive and adaptive personality traits, such as agreeableness, empathy, forgivingness, and generosity (McCullough et al., 2002) as well as trust and altruism (Wood et al., 2010), while scoring lower on envy and materialism (see McCullough et al., 2002). Overall, people with dispositional gratitude seem better able to regulate their responses in the face of unfair offers.

Dispositionally grateful people more consistently recognize the presence of benefits as gifts offered by others, even when such offers are relatively small. Thus, they are more likely to recognize the benefit of receiving $1, $2, or $3 in even an unequal offer from a human proposer. With this positive appraisal, gratitude likely down-regulates or countervails negative emotions associated with violation of the fairness norm in the UG task, resulting in greater acceptance of unfair offers.

Trait gratitude could motivate people to engage in positive behavior that benefits both players involved in the game. People with high dispositional gratitude are more empathic, forgiving, and generous (McCullough et al., 2002; Witvliet et al., 2018b), as well as more hopeful and happy (Witvliet et al., 2018a) and more likely to make more positive appraisals of a situation (Wood et al., 2008). For example, they are more generous in their interpretations, and they may be able to see benevolent possibilities, such as that the proposer who made an unfair offer might be in need of money rather than intentionally violating the social norm of fairness. Or, they may see that establishing a prosocial relationship with the proposer might outweigh emotional and social consequence of punishing them. Such positive interpretations of social situations would allow people with high trait gratitude to reason that a small amount of profit is better than nothing in the UG, leading them to accept more unfair offers.

Our results link trait gratitude to effective economic decision-making and are broadly consistent with recent neuroimaging studies which show that the neural mechanisms involved in effective economic decision-making are also implicated in mediating gratitude. Recent neuroimaging studies have shown that ratings of gratitude are associated with greater activations in a brain region of the medial prefrontal cortex (MPFC) that encompassed the peri-genual anterior cingulate cortex (ACC) and the ventral and dorsal MPFC (Fox et al., 2015; Kini et al., 2016; Yu et al., 2016). These brain areas have also been associated with the reward system involved in computing and updating the values of social and nonsocial behavior (Schultz, 2015), as well as fairness and economic decision-making (Tabibnia and Lieberman, 2007). Thus, it is possible that individual differences in gratitude may weigh in computing and updating the value of social behavior and contribute to making more optimal economic decision-making. However, neuroimaging evidence will be necessary to further clarify the neural mechanisms that underlie the relationship between gratitude and economic decision-making.

There are some limitations of the current research. Participants completed the GQ-6 (McCullough et al., 2002) at the conclusion of the study in an effort to ensure that participants randomly assigned to the control condition were not primed with gratitude statements. Thus, it is possible that the effects attributed to trait gratitude might in part reflect the effect of the state gratitude induction, which elevated state gratitude, although it did not result in more acceptance of unfair offers. The lack of state gratitude effects on UG task in the present study may appear to contradict previous research in which people with higher state gratitude showed reduced impatience and chose larger long-term benefits over small immediate benefits in the delay discounting task (DeSteno et al., 2010). In the current study, however, a momentary elevation of state gratitude may not have been potent enough to counter negative emotions associated with violation of the fairness norm. Rather, trait gratitude – which is associated with an entrenched dispositional pattern across a variety of situations, benefactors, and benefits – was potent enough to be associated with the UG task. In other research, when participants employed an emotion appraisal regulation technique to control negative emotions associated with unfair offers, they accepted more unfair offers (van’t Wout et al., 2010). In the context of an interpersonal injustice, benefit-focused reappraisals were associated with gratitude, forgiveness, down-regulation of negative emotions, and cardiac regulation. Unfortunately, we were not able to measure emotional conditions associated with unfair offers because this would have disrupted game play (van’t Wout et al., 2010). The non-significant state result in the current study may be due to lack of power. It may also be that a different type of experimental induction could have a stronger effect.

Future research that aims to develop gratitude and test effects on decision-making can draw on a large body of research. Davis et al. (2016) conducted a meta-analysis of 26 studies in which participants were randomly assigned to gratitude interventions. Findings indicated that participants experienced psychological well-being benefits from gratitude interventions in comparison to non-treatment measurement conditions (*k* = 5, *d* = 0.31) or inert matched activities (*k* = 18, *d* = 0.14), yet not in comparison to other positive psychological conditions (*k* = 9, *d* = −0.03). A more recent gratitude writing intervention induced hope and happiness relative to a control writing condition (Witvliet et al., 2018a). To the extent that affect enhancement mitigates retaliation as the rejection of unfair offers, these studies offer a range of gratitude interventions that could be tested to assess their capacity to overcome the tendency to reject unfair offers at cost to oneself.

Future research could also adapt interventions from the forgiveness literature that generate gratitude. One promising approach is to design a gratitude-oriented benefit-finding reappraisal that has been effective in identifying even small benefits in negative social situations such as offenses (e.g. Witvliet et al., 2010). This could be relevant and effective for testing the UG task because it involves overcoming the unjust inequity within unfair offers. Benefit-finding may help economic game participants enact better decisions (accepting even unfair offers in the UG task) with outcomes that also benefit oneself.

## Conclusion

The current experiment examined the roles that trait and state gratitude play in making an optimal economic decision when respondents’ sense of fairness is violated. High trait, but not momentary state, gratitude was associated with accepting unfair offers, which benefit both parties involved. Thus, it appears that the disposition to be grateful across situations, givers, and benefits plays an integral role in overcoming the typical response to violation of the fairness norm in order to make the most economically effective decision.

## Data Availability Statement

The datasets presented in this study can be found in online repositories. The names of the repository/repositories and accession number(s) can be found at: https://osf.io/njrpy/.

## Ethics Statement

The studies involving human participants were reviewed and approved by Azusa Pacific University IRB committee. The patients/participants provided their written informed consent to participate in this study. Written informed consent was obtained from the individual for the publication of any potentially identifiable images or data included in this article.

## Author Contributions

GP designed the experiments and collected data. GP analyzed the data and wrote the manuscript with critical edits from Cv-W, JB, and BM. All authors contributed to the article and approved the submitted version.

### Conflict of Interest

The authors declare that the research was conducted in the absence of any commercial or financial relationships that could be construed as a potential conflict of interest.

## Footnotes

^1^We used a sample size from previous research with a similar hypothesis (DeSteno et al., 2012). The data are available online at: https://osf.io/njrpy/.

^2^We also tested whether individuals with higher trait gratitude would produce higher state gratitude after the gratitude induction. Participants were divided into two groups – high or low trait gratitude – based on the median split of trait gratitude scores (GQ-6). We conducted a 2 (Trait gratitude: high, low) × 2 (Induction Condition: neutral, grateful) × 2 (Measured Time: before the induction, after the induction) mixed analysis of variance, with Mixed Time factor being repeated. There was no significant three-way interaction between induction condition and measured time, *F*(1, 65) = 3.35, *p* = 0.07, !$\eta_{p}^{2}$ = 0.05. In fact, individuals with high trait gratitude showed significantly increased state gratitude after the induction, *t*(18) = ‒3.01, *p* < 0.01, *d* = 0.36, and individuals with low trait gratitude showed the same effect, *t*(15) = ‒4.39, *p* < 0.01, *d* = 0.68.

^3^We also examined the relationships between trait gratitude and the acceptance rates of unfair offers after controlling for changes in state gratitude. The relationships between trait gratitude and the acceptance rates of unfair offers were subjected to a first-order partial correlation in order to explore the relationships after controlling for the effect of changes in state gratitude. The first-order correlations were found to be statistically significant, *r* = 0.26, *p* = 0.04 for $9:$1; *r* = 0.27, *p* = 0.03 for $8:$2; *r* = 0.27, *p* = 0.03 for $7:$3, indicating that the relationships between trait gratitude and the acceptance rates of unfair offers exist after controlling for the effect of state gratitude.
